# A feces‐filled non‐inverted ileal pseudodiverticulum presenting as a pedunculated polyp successfully treated by traction‐assisted endoscopic submucosal dissection

**DOI:** 10.1002/deo2.139

**Published:** 2022-06-14

**Authors:** Taisuke Inada, Yorinobu Sumida, Eikichi Ihara, Chikako Yoshitake, Akito Ohkubo, Naru Tomoeda, Shohei Hamada, Yoichiro Iboshi, Makoto Nakamuta, Naohiko Harada

**Affiliations:** ^1^ Department of Gastroenterology and Clinical Research Institute National Hospital Organization Kyushu Medical Center Fukuoka Japan; ^2^ Department of Medicine and Bioregulatory Science Graduate School of Medical Sciences Kyushu University Fukuoka Japan

**Keywords:** diverticulum, endoscopic resection, feces, ileum, polyp

## Abstract

A 68‐year‐old man was referred to our hospital for endoscopic treatment of colon polyps detected at a local clinic. Colonoscopy revealed not only classical adenomatous polyps in the transverse and sigmoid colon but also an atypical pedunculated polyp in the terminal ileum with the head of the lesion moving back and forth through the ileocecal valve. Based on the endoscopic findings, the pedunculated polyp was diagnosed as a non‐epithelial tumor of the ileum. However, traction‐assisted endoscopic submucosal dissection was performed because of the high risk of intestinal intussusception or obstruction. Histopathological analysis of the resected specimen revealed that the pedunculated polyp was a non‐inverted ileal pseudodiverticulum filled with feces. We report the first case of a feces‐filled non‐inverted pseudodiverticulum presenting as a pedunculated polyp successfully treated by traction‐assisted endoscopic submucosal dissection.

## INTRODUCTION

Ileal diverticulum is a relatively rare condition. The most frequently occurring type of ileal diverticulum is Meckel's diverticulum, while non‐Meckelian ileal diverticula are extremely rare.^1^, ^2^ Pseudodiverticula of the small intestine reportedly occur in only 1%–5% of the general population^3^ and are thought to result from abnormalities of the smooth muscle and intermuscular plexus. There are reports of ileal diverticula causing intestinal obstruction and intussusception^4^; these complications require surgical treatment. We experienced a case in which an ileal pseudodiverticulum filled with feces presented as a pedunculated polyp. This is the first case report of a feces‐filled non‐inverted ileal pseudodiverticulum successfully treated endoscopically.

## CASE REPORT

**FIGURE 1:**
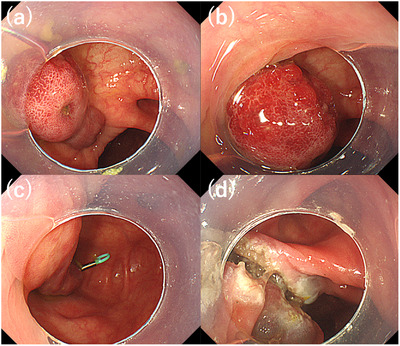
Endoscopic submucosal dissection of the ileal pedunculated polyp. (a, b) Endoscopic images showing the ileal pedunculated polyp with a small concavity (a) and reddish mucosa (b) on the polyp head. No mucosal atypia is evident. (c) Endoscopic image showing the traction of the ileal pedunculated polyp attained by applying a countertraction clip attached to the stalk of the polyp. (d) Endoscopic image showing both the submucosal and muscular layers in the post‐endoscopic submucosal dissection ulcer floor

A 68‐year‐old man had undergone a colonoscopy at a local clinic after receiving a positive fecal occult blood test result. Because colon polyps were detected, he was referred to our hospital for endoscopic treatment. He had been taking amlodipine besylate hydrochloride, cilostazol, and clopidogrel sulfate for hypertension and arteriosclerosis obliterans. There were no abnormal findings on routine blood tests. Colonoscopy revealed not only classical adenomatous polyps in the transverse and sigmoid colon but also an atypical pedunculated polyp in the terminal ileum with the head of the lesion moving back and forth through the ileocecal valve. The lesion had a small depression on the lateral side of the head (Figure 1a). The head of the lesion was covered with congested, reddish, and swollen villous mucosa, but showed no structural atypia (Figure 1b). Based on the endoscopic findings, the lesion was diagnosed as a non‐epithelial tumor of the ileum. Because the lesion was moving back and forth between the ileum and cecum through the ileocecal valve, there was a high risk of intestinal intussusception or obstruction. Therefore, endoscopic treatment with endoscopic submucosal dissection (ESD) was performed after obtaining informed consent from the patient.

**FIGURE 2:**
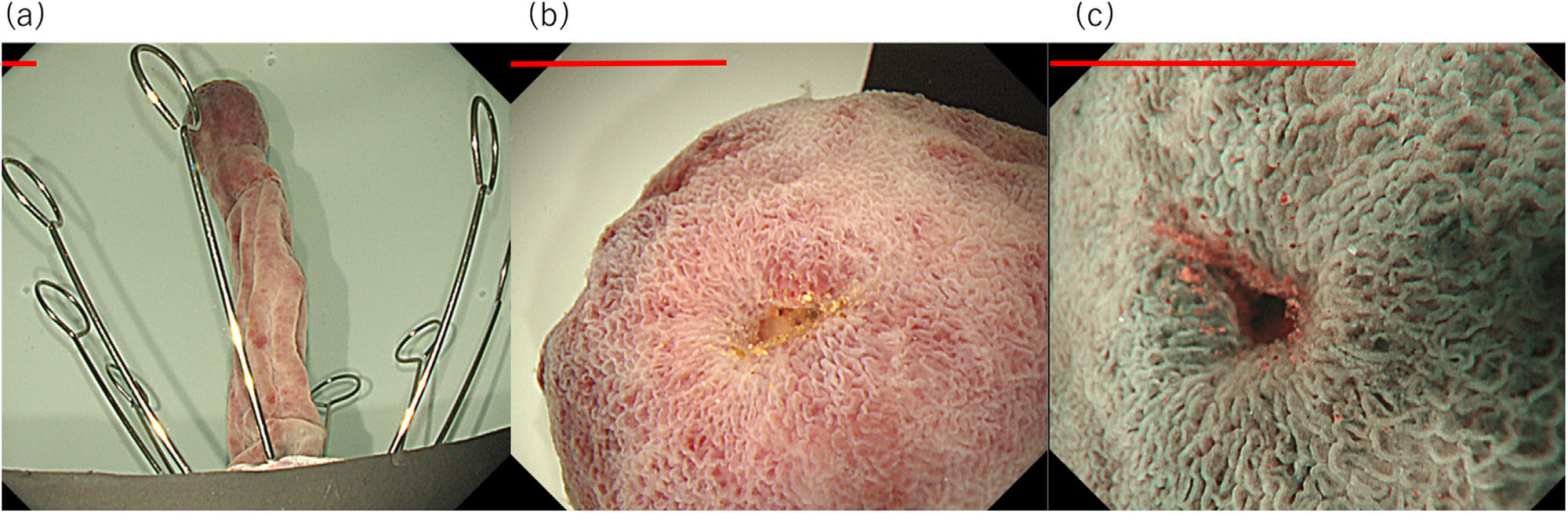
Endoscopic images of the resected ileal pedunculated polyp specimen. (a–c) Endoscopic images showing (a) an overall view of the lesion, (b) a close‐up view of the polyp head with white light, and (c) a magnified view of the polyp head with narrow‐band imaging. The edge of the opening of the polyp head is composed of normal mucosa. Scale bar: 0.5 cm

The base of the lesion was pulled out and fixed in the colon by applying traction towards the cecum using a countertraction clip (S‐O clip; Zeon Medical Inc.) that was placed at the base of the lesion (Figure 1c,d). A mucosal incision was created at the base of the lesion with an ESD knife (Dual Knife; Olympus Co. Ltd.), followed by submucosal dissection to achieve en bloc resection (Figure 2a–c). The ESD‐associated ulcer floor was completely closed with clips (Zeoclip; Zeon Medical Inc.). There were no procedure‐related complications. In accordance with advice from the prescribing physician, clopidogrel was not discontinued, while cilostazol was discontinued only on the day of ESD and the day after ESD. The patient was discharged 2 days after the endoscopic treatment.

**FIGURE 3:**
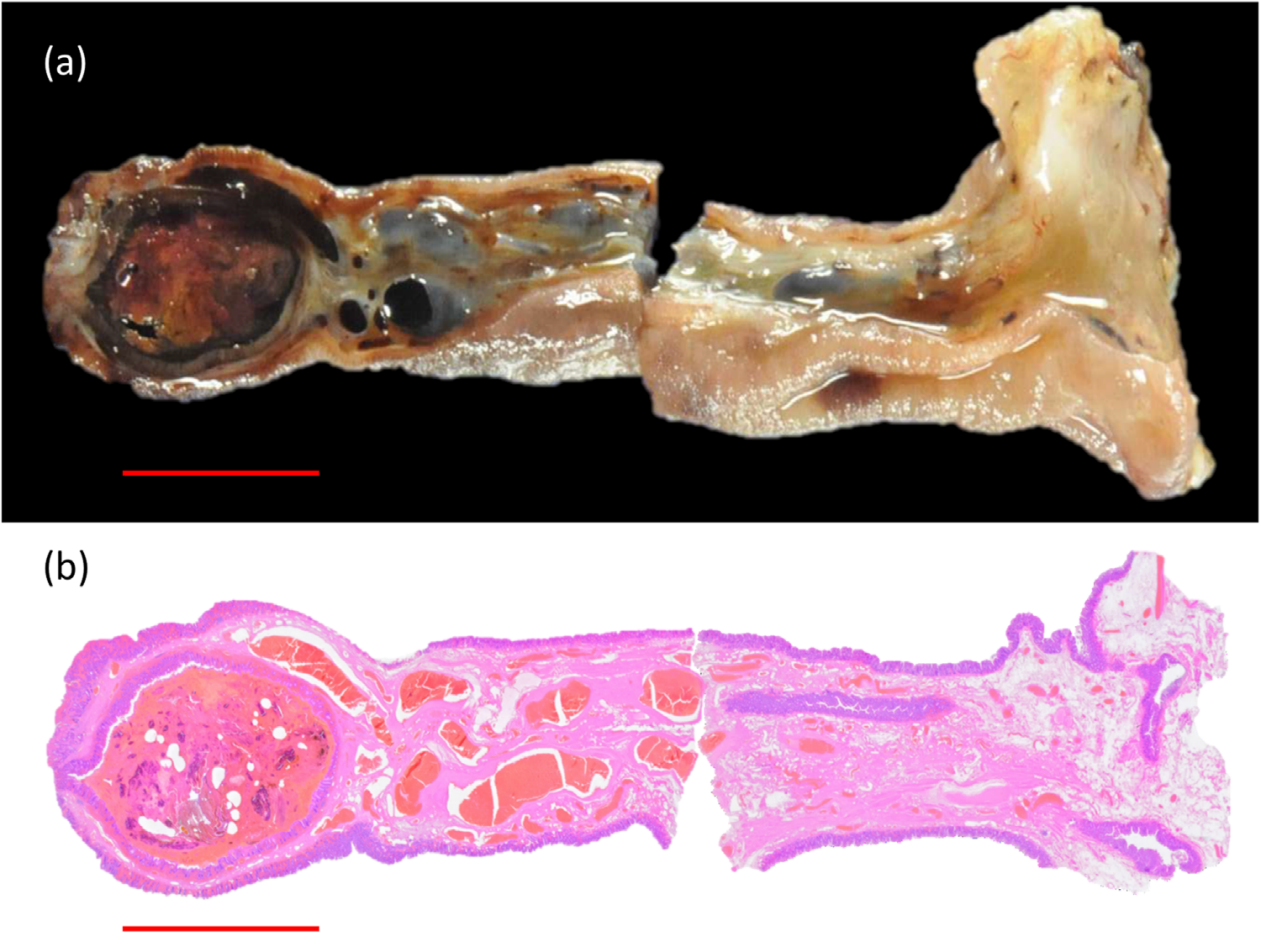
Pathological findings of the resected ileal pedunculated polyp specimen. (a) Macroscopic pathological image showing that the head of the polyp is filled with feces and is 10 mm in size. No striae are seen at the base. (b) Macroscopic pathological image with hematoxylin and eosin staining showing that the polyp head is filled with feces inside the inverted mucosa. The base is formed by submucosa with cystically dilated and congested blood vessels and normal mucosa. Scale bar: 1 cm

Histopathological analysis of the resected specimen revealed that the head of the lesion was a pseudodiverticulum that was filled with feces (Figure 3a,b). The base of the lesion was composed of mucosa, muscularis mucosae, and submucosa, but did not contain the muscle layer or serosa. The lesion was diagnosed as a non‐inverted ileal pseudodiverticulum filled with feces. The depressed area on the side of the head of the lesion was an orifice of the pseudodiverticulum. No ectopic gastric tissue or pancreatic tissue was detected, and there was no evidence of Meckel's diverticulum.

## DISCUSSION

Ileal diverticula are relatively rare, especially non‐Meckelian ileal diverticula. The lesion in the present case was located at the terminal ileum, and histopathological examination revealed that it contained no ectopic gastric or pancreatic tissue and no intrinsic muscular layer or serosa. Based on these findings, the lesion was diagnosed as an ileal pseudodiverticulum.

In the present case, the pseudodiverticulum was filled with feces and the morphology of the pseudodiverticulum itself was preserved, which is different from an “inverted diverticulum”. We hypothesized that the mechanism of the pedunculated polyp in the present case was similar to that of a colonic muco‐submucosal elongated polyp^5^ or pedunculated submucosal tumor.^6^ After the ileal pseudodiverticulum with a small orifice was filled with feces, it rose above the surrounding area and developed into a pedunculated structure because of the high degree of wall extensibility, active peristalsis, and high luminal pressure of the terminal ileum. There are no reports of similar lesions in the colon, although feces‐filled diverticula are common. This may be because colonic diverticula usually have large orifices that allow easy evacuation of feces, and any feces‐filled colonic diverticula that develop are rarely elevated because of the low degree of wall extensibility of the colon compared with the ileum.

It would have been difficult to diagnose the lesion as a pseudodiverticulum before surgery. At the time of ESD treatment, the lesion was thought to be a non‐epithelial tumor with no evidence of malignancy. The lesion was resected to obtain a definitive diagnosis and to prevent it from causing intestinal obstruction or intussusception. Endoscopic resection is reportedly useful for the removal of benign epithelial tumors, polyps, and intramucosal carcinomas of the small intestine.^7^ In the present case, endoscopic mucosal resection was initially planned but was abandoned because the polyp head entered the ileum through the Bauhin valve, making snaring difficult. Because non‐epithelial tumors are small and can be resected endoscopically, we opted to perform minimally invasive ESD. During ESD performed to resect lesions arising from the terminal ileum, it is useful to pull the target lesion and fix it on the colon side. In the present case, we were able to safely resect the lesion by applying traction towards the cecum with S‐O clips. This traction‐assisted ESD provided direct visualization of the inner part of the cutting line and enabled accurate dissection of the submucosal layers without damaging the muscular layers.^8^

We reported the first case of a feces‐filled non‐inverted ileal pseudodiverticulum that was successfully treated endoscopically.

## CONFLICT OF INTEREST

The authors declare that they have no conflict of interest.

## FUNDING

None.

## ETHICS STATEMENT

All procedures were performed in accordance with the ethical standards of the Declaration of Helsinki and its later amendments.
